# The Outcome of Neurorehabilitation Efficacy and Management of Traumatic Brain Injury

**DOI:** 10.3389/fnhum.2022.870190

**Published:** 2022-06-22

**Authors:** Miyamoto Akira, Takata Yuichi, Ueda Tomotaka, Kubo Takaaki, Mori Kenichi, Miyamoto Chimi

**Affiliations:** ^1^Faculty of Rehabilitation Sciences, Nishikyushu University, Kanzaki, Japan; ^2^Faculty of Human Science, Hokkaido Bunkyo University, Eniwa, Japan; ^3^Division of Physical Therapy, Department of Rehabilitation, Faculty of Health Science, Kumamoto Health Science University, Kumamoto, Japan; ^4^Omote Orthopedic Osteoporosis Clinic, Toyonaka, Japan; ^5^Department of Occupational Therapy, Faculty of Health Science, Aino University, Ibaraki, Japan

**Keywords:** traumatic brain injury, neurorehabilitation, treatment, curing, neuroscience

## Abstract

For public health professionals, traumatic brain injury (TBI) and its possible protracted repercussions are a significant source of worry. In opposed to patient neurorehabilitation with developed brain abnormalities of different etiologies, neurorehabilitation of affected persons has several distinct features. The clinical repercussions of the various types of TBI injuries will be discussed in detail in this paper. During severe TBI, the medical course frequently follows a familiar first sequence of coma, accompanied by disordered awareness, followed by agitation and forgetfulness, followed by return of function. Clinicians must be aware of common medical issues that might occur throughout the various stages of neurorehabilitation, for example, posttraumatic hydrocephalus, paroxysmal sympathetic hyperactivity and posttraumatic neuroendocrine disorders, at each step of the process. Furthermore, we address problems about the scheduling of various rehabilitation programs as well as the availability of current data for comprehensive rehabilitative neuropsychology techniques.

## Introduction

Research evidence indicates that approximately 40 percent of people who were admitted as a consequence from a mild to severe traumatic brain injury (TBI) experience protracted impairment, with incidence rates ranging from 3.2 to 5.3 million people in the United States (US), or even more than 1.1% of the total residents of the US (Zaloshnja et al., 2008; Agimi et al., 2021). In 2020, there were about 64,000 TBI-related fatalities in the US. Every day, over 176 people die as a result of TBI (CDC, 2022). In Japan, TBI accounted for only 9.3% of 1,160,000 traffic accident injuries. However, of the 9,000 deaths in these data, 50.3% of them were in fact due to TBI (Kimura, 2003). TBI mortality in japan decreased from 29.5 to 14.2% in the last decade, which is comparable to rates recorded in other countries (Hosomi et al., 2022). Despite a paucity of data on the economic effect of this issue, estimates for the United States put the yearly cost at more than 221 billion dollars (Laskowitz and Grant, 2016), with estimates from the Centers for Disease Control and Prevention (CDCP) being more modest at 56 billion dollars. The average cost per case ranged from $33,284 to $35,954 for mild TBI and from $25,174 to $81,153 for significant TBI (Humphreys et al., 2013). Inadequate consideration of indirect costs may result in an overestimation of the overall effect (caregivers and relatives provide care and assistance). As a result, not only from the perspective of the individual affected, as well as from a purely economic one, it looks fair to assist affected people in their recovery and return to independence *via* the use of an optimum rehabilitation time.

The field of neurorehabilitation after TBI is vast and multifaceted, encompassing everything from initial rehabilitation of persons with reduced consciousness through assistance and companionship as patients reintegrate into their social and occupational situations. The assessment of relevant current literature covering the diverse components of TBI restoration in a balanced manner is thus not viable for a brief review paper on this subject matter. Consequently, we concentrated on inpatient rehabilitation and certain particular themes that are medically significant in neurorehabilitation of TBI, and we highlighted the differences between inpatient rehabilitation and treatment for other types of brain lesions. The first part of our review focused on the TBI pathophysiology, management and clinical features and lesion variation to clear a brief overview to the readers about the TBI. Next, we elaborate various critical features for TBI rehabilitation and the admittance of TBI patients to institutions. Finally, we elaborate the impact of timing, intensity, duration and rehabilitation programs on TBI rehabilitation outcomes.

To search the literatures, we used PubMed and Web of Sciences using the terms “Neurorehabilitation,” “Traumatic Brain Injury,” and “disorders of consciousness,” “cognitive rehabilitation,” “neuropsychological rehabilitation,” and “management of Traumatic Brain Injury.” We included only English literatures and excluded literatures from other languages. Moreover, we also excluded abstracts and conferences.

## Traumatic Brain Injury Pathophysiology and Management

The first stage after a TBI is characterized by tissue deterioration, poor autoregulation of cerebral blood flow (CBF), and metabolic abnormalities. As with ischemia, this scenario may result in the accretion of lactic acid, an increase in the permeability of the cell membrane, and eventually edema. Due to anaerobic metabolism’s inability to satisfy the brain’s requirements, adenosine triphosphate (ATP) reserves become depleted, leading in the loss of the ATP-dependent membrane The 2nd step of this cascade is characterized by prolonged membrane depolarization, excitotoxicity (high flow of excitatory neurotransmitters such as aspartate and glutamate), and stimulation of voltage-dependent Na+ and Ca++ channels. Following Ca and Na entry, proteases, lipid peroxidases, and phospholipases are stimulated, commencing the apoptotic pathway, ultimately resulting in membrane breakdown and cell death (Picetti et al., 2021).

## Traumatic Brain Injury and Oxidative Stress

Some other kind of cell death that occurs quickly oxidative stress after a TBI, which is characterized by the buildup of both reactive oxygen (ROS) and nitrogen species (NS) (Dasuri et al., 2013). Lipoperoxidation of the cellular membrane occurs when ROS levels rise, resulting in mitochondrial and oxidizing protein malfunction, which may affect the shape of membrane pores (Mutinati et al., 2014). TBI stimulates microglia cells, which produce astrocytes, as well as proinflammatory cytokines, which may enhance the expression of neurotrophic factors generated from the brain. These, in turn, aid and guide axon healing, boost cell formation, and promote long-term neuronal survival by preventing cell death (Wu et al., 2014). Additionally, astrocytes modulate extracellular glutamate levels, therefore reducing neuronal and other cell excitotoxicity caused by glutamate (Kumar and Loane, 2012). The observed pathophysiological variability in patients having TBI can be explained by the kind, intensity, and site of the initial damage, as well as by gender, age, genetics, and medication use (Margulies and Hicks, 2009).

## Medical Management of Traumatic Brain Injury With Drugs

At the moment, there is no drug available to prevent nerve injury or to enhance nerve recovery after a TBI. The fundamental purpose of the intensive care unit is to avoid further brain damage. The term “primary insult” refers to the original brain damage, whereas “secondary insult” refers to any future development that may result in neurological harm. For instance, a wounded brain is more sensitive and fragile to blood pressure drops that are normally easily tolerated. Attempting to maintain normal or slightly increased blood pressure levels is one strategy to prevent secondary insults.

Secondary damage is mostly caused by the neuroinflammatory process, which is characterized by prolonged microglial stimulation, astrocyte activation, the production of oxidative stress and pro-inflammatory cytokines. It has been noted that it is critical to initiate treatment measures quickly after TBI, preferably within 4 h of the damage, in order to get the most promising neuroprotective effect (Sullivan et al., 2011). Minocycline, a tetracycline derivative, is particularly effective in reducing apoptotic and inflammatory manners in a variety of models of central nervous system (CNS) disease (Orsucci et al., 2009). Along with pharmaceutical therapies for TBI, prospective, creative innovations based on preclinical results use biologics (for instance, DNA, gene therapy, eRNA, microRNA, antagonists stem cells, peptide therapy, peptides, and exogenous growth factors) (Mouhieddine et al., 2014). Neuronal and mesenchymal stem cell therapies have the potential to be neuroregenerative and neurorestorative (Liao et al., 2015).

Many people with mild to severe head injuries are immediately transferred from the emergency area to the operation room. Often, surgery is undertaken to remove a big hematoma. Following surgery, these individuals are monitored in an intensive care unit (ICU).

## Clinical Features and Lesion Variation

### Lesion Variability

**FIGURE 1:**
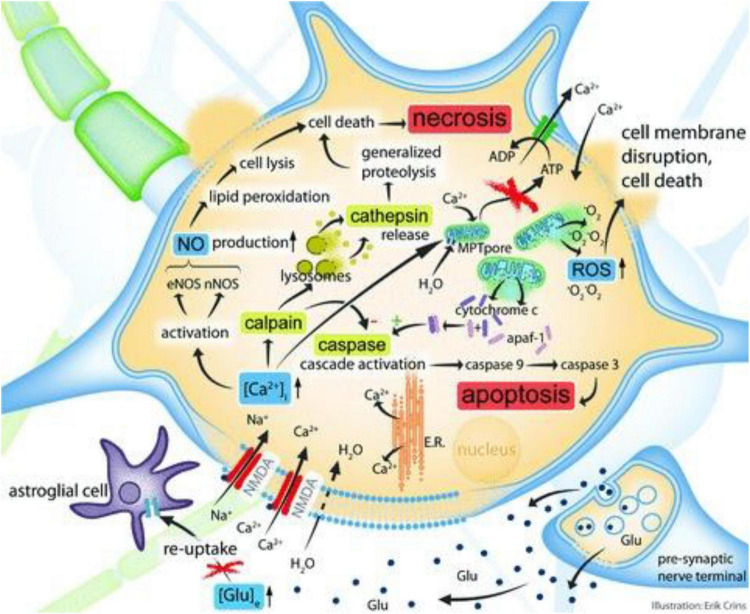
Simplified cellular and molecular pathophysiological pathways after localized TBI. The figure is used to accompany the record provided in the actual section. In summary, elevations in extracellular glutamate cause a supraphysiological Ca2 + influx, which then activates some intracellular cascades acting in tandem (four are shown). Upregulation of the calcium-dependent enzymes eNOS and nNOS results in increased nitric oxide generation, which finally results in necrosis of cells and lipid peroxidation. The activity of calpain (a cystein protease) is also increased as a consequence of increased intracellular calcium, eventually culminating in cellular necrosis pathways. This process is facilitated by cathepsin release and lysosomal membrane disruption. Developments in intracellular calcium that cause mitochondrial calcium overload enhance the permeability of the mitochondrial membrane. As a consequence, reactive oxygen species (ROS) and the cytochrome-c protein are discharged into the cytoplasm. Cytochrome-c interacts with the apoptosis activating protein-1 (apaf-1), initiating the caspase pathway that induces apoptosis. Glu, glutamate; [Glu]e, extracellular glutamate concentration; NMDA, *N*-methyl-D-aspartate aspartic acid; E.R., endoplasmatic reticulum; [Ca2 + ]i, intracellular Ca2 + concentration; nNOS, neuronal NOS; eNOS, endothelial NOS; MPT, membrane permeability transition; ATP, adenosine triphosphate; O2 is an abbreviation for oxygen radical. Reproduced with permission from Andriessen et al. (2010).

One may expect that neurorehabilitation after every kind of brain damage, regardless of its cause (ischemic, hemorrhagic, traumatic, or hypoxic), would be identical. Though, this is not the case. In reality, however, this is not true since the methods of brain injury and localization of the lesions are highly distinct amongst the various categories of people. Ischemic stroke causes injury to the brain, which causes impairment and localized practically anywhere in the brain, although it may show certain areas of preference. TBI, on the other hand, frequently results in bi-hemispheric contusions and abnormalities, certain of which are extremely confined (hematomata in the parenchyma), while others are more widespread (hematomata in the subarachnoid, subdural, and epidural spaces, respectively). As a result, broad damage to cell bodies and axons are common (Adams et al., 1989; Andriessen et al., 2010; Dasuri et al., 2013; Humphreys et al., 2013; Mutinati et al., 2014; Laskowitz and Grant, 2016; Ma et al., 2016; Picetti et al., 2021; Hosomi et al., 2022), the latter of which is specified to as diffuse axonal injury (DAI) (Adams et al., 1989; Andriessen et al., 2010; Dasuri et al., 2013; Humphreys et al., 2013; Mutinati et al., 2014; Laskowitz and Grant, 2016; Ma et al., 2016; Picetti et al., 2021; Hosomi et al., 2022). These forms of injury are trailed by significant diffuse edema of the brain, which results in a variety of modifications at the cellular and metabolic alterations (Andriessen et al., 2010). Some key changes are highlighted in the Figure 1.

**FIGURE 2:**
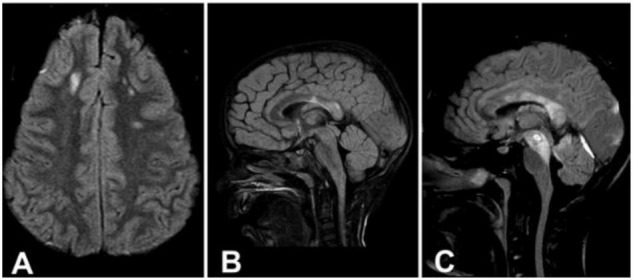
**(A)** Axial image demonstrating signal intensity changes in the lobar white matter (Stage 1). **(B)** Sagittal image demonstrating signal intensity changes in the splenium of the corpus callosum (Stage 2). **(C)** Sagittal image demonstrating signal intensity changes in the rostral brainstem (Stage 3). Reproduced with permission from Skandsen et al. (2010).

Even in the case of cognitive impairment, standard magnetic resonance imaging (MRI) arrangements often result in an underestimation of the extent of the impairment (Kinnunen et al., 2011). One year after the injury, a Norwegian research of 106 individuals who had imaging within 4 weeks of their TBI found an association between the severity of DAI and poorer Glasgow outcome scale-extended scores (Skandsen et al., 2010). Figure 2 shows the three stages of DAI.

### To Clinical Patterns

Depending on the clinical manifestation of the lesion, localized ischemia or hemorrhage, for example, may be distinguished. A particular predisposition to frontotemporal lesions has been seen in concussion-related injury, which often results in difficulties in attention, executive function, and memory along with possibly subtle deficiencies in in moral and social behavior (Bigler, 2007). As a result of the disruption of inherent connection networks at a huge scale, specifically frontal and limbic connections, DAI is more frequently related at the acute stage, with diminished awareness (Skandsen et al., 2010) and cognitive dysfunction in the subacute and chronic phases (Scheid et al., 2006; Sharp et al., 2014), which is likely due to the disruption of massive scale inherent connection networks with precise disruptions of limbic and frontal influences.

Nonetheless, the difference between focal and diffuse injuries is rather random, since they are frequently found in the same patient and are likely to be seen in more than 50 percent of TBI patients, as indicated by an MRI research (Lee and Newberg, 2005). TBI-induced cognitive impairment is frequently supplemented by behavioral alterations for example lack of inventiveness, irritation, and deprived expressive regulation, among other symptoms of the disorder. It is worth noting that, when compared to cognitive or behavioral disorders, as well as the result of a stroke, persistent motor weakness following a TBI is quite rare (Jang, 2009). More frequently found in individual with milder types of TBI, symptoms for example headache, dizziness, exhaustion, sleep difficulties, and balance issues are typical complaints that endure for a long amount of time in a high percentage of patients (Ponsford et al., 2012, 2014). In addition, psychological discomfort, anxiety disorders, depression, and drug addiction have been found in one study to be quite common up to 75% of patients were impacted (Alway et al., 2016).

The natural history of TBI recovery follows a distinct sequence of severe TBI comprises of a period of reduced awareness, that may be characterized by paroxysmal sympathetic hyperactivity (PSH), then a time of posttraumatic agitation (PA) and/or misperception with amnesia, and lastly a phase of post-confusional function retrieval, which is characterized by a variety of emotional, cognitive, sensorimotor and behavioral, and disturbances (Povlishock and Katz, 2005). Depending on the specific path of retrieval and the exact time in repossession at which rehabilitation is undertaken, one may be faced with a variety of medical issues. We shall go into further depth about some of these features of TBI therapy in the next sections.

In addition, moderate-to-severe TBIs are frequently associated with polytraumatic damages, which include multi-organ damage, burns, fractures, and obstruction. So the wide range of comorbid illnesses and brain trauma abnormalities itself lead to a highly distinct display of physical, behavioral cognitive, and psychosocial problem in individual who have experienced a TBI, necessitating the use of a rehabilitation team that is specifically trained in the neurorehabilitation of TBI.

## What Medical Features Are Particularly Important in Traumatic Brain Injury Rehabilitation?

### Disorders of Consciousness

Serious TBI can have long-term consequences in protracted spells of coma or unconsciousness. After a period of time, post-traumatic coma (defined by Posner and Plum as a phase of «unarousable unresponsiveness» with closed eyes constantly and no medical or electroencephalographic reaction to ambient or innate motivation frequently progresses to states with increased consciousness or stimulation, like as minimally conscious state (MCS), vegetative state (VS), and unresponsive wakefulness syndrome (UWS), which are all referred to as Disorders of Consciousness (DOC) (Plum and Posner, 1982).

In the state of severe TBI, patients do not exhibit symptoms of conscious activity, but they do display impulsive eye opening and sign of cycle of sleep and wakefulness in their EEG, because they are not required artificial breathing in most cases (Giacino and Whyte, 2005). European task group on DOC in 2010, suggested the acronym UWS with the purpose of change the word VS, which was typically associated with negative connotations (Laureys et al., 2010). American Neurological Association define MCS is “a condition of severely altered consciousness in which minimal but definite behavioral evidence of self or environmental awareness is demonstrated.” It must only utilized if there is perfect and repeatable proof of non-reflexive behavior (Giacino et al., 2002).

Even in the 21st century, Clinical assessment continues to be the gold standard for evaluation and DOC assessment. The Coma Recovery Scale-Revised (CRS-R) is a tool that is frequently utilized in this setting for evaluation. It concentrate on and assesses evidence of conscious awareness in relation to physical and verbal behavior, and as a result, it allows for the identification of variations in the medical condition (Giacino et al., 2004). In recent times, further scales have been produced to aid DOC assessment, i.e., Motor Behavior Scale (MBS) and the DOC Scale (DOCS-25) (Mallinson et al., 2016; Pignat et al., 2016), which are examples of scales that have been emerged and tested in DOC populations.

According to a study conducted in a Norwegian population, two percent of patients were in MCS or VS 3 months after severe TBI, and fewer than one percent of patients were in a condition of DOC after 1 year (Løvstad et al., 2014). Both situations have the potential to remain or to progress into higher degrees of awareness, which are often characterized by the ability to engage and communicate with one’s surroundings. The likelihood of emerging from these states and regaining functional recovery succeeding a TBI is often inversely connected to the length of the DOC after TBI (Whyte et al., 2005; Katz et al., 2009; Hammond et al., 2019), but not always (Lammi et al., 2005). In the first 6 months after an accident, a substantial majority of individuals who will improve will do so (Choi et al., 1994). In spite of this, some examples have revealed that development from DOC may happen long beyond the 1st year subsequently a TBI (Levin et al., 1991; Childs and Mercer, 1996). In a research of extremely serious TBI individuals, the writers reviewed that individuals with MCS or who were anaesthetized 03 weeks after the damage had a enhanced prognosis than patients who were in UWS or who were in coma (Godbolt et al., 2013). The findings are consistent with those of Katz et al. (2009), who found that most of their individual with MCS (72 percent) or posttraumatic amnesia/confusional state (CS/PTA, 58 percent) developed from these states in a study of 36 patients with DOC. Furthermore, the length of MCS was shown to be linked with the speed with which CS/PTA recovered. Patients who had been in VS for more than 8 weeks did not ever leave the CS/PTA level of care. A previous review study, which re-examined information from 434 individuals, found that development from VS happened approximately half of the patient, between 6 and 12 months in 6 percent, and away from 12 months in just 1.6 percent of the patients (Multi-Society Task Force on PVS, 1994).

In a recent study, data from a 110-person population, individuals with chronic pain because of TBI) who were tracked for 10 years were reported. By 1 year after the injury, a majority of the participants had attained near-maximal recuperation. However, a subset of patients who had to wait a long time to get out of DOC (a statement that succeeds more than 4 weeks after a TBI) nonetheless shown considerable useful recovery between 2 and 10 years after the injury (Hammond et al., 2019). The majority of individuals with UWS/VS or MCS who recover consciousness has been reported to vary substantially in the literature, owing to the variability of descriptions, the varying time intervals at which patients were included in the research, and the fact that many research do not have long-term follow-up. The results of certain studies might be skewed because of selection bias [for instance, Katz’s inhabitants (Katz et al., 2009) consisted only of individual who were hospitalized to an acute rehabilitation hospital’s specialist slow-to-recovery brain injury program]. Finally, Hammond’s evidence (Hammond et al., 2019) illustrates that functional improvement may occur over a lengthy period of time after a stroke. As a result, predicting individual outcomes might be difficult.

Schnakers and Monti (2017) did a thorough literature analysis and found that the majority of DOC therapy reports pertain to single patients or short case sequence, with just a few randomized controlled trials being published. The most recent known data comes from a medication study including amantadine. In a 4-week randomized, multicenter research, 184 patients with MCS or VS were randomly allotted to receive either amantadine (in increasing dosages up to 200 mg two times a day) or a palliative treatment. Patients with VS or MCS who were treated with amantadine showed faster useful reclamation on the Disability Rating Scale (DRS) as related to individuals who were treated with placebo (Giacino et al., 2012).

It has been noted in few situations that zolpidem had a paradoxical effect, resulting in a brief recurrence of consciousness depression. Two bigger placebo-controlled experiments (Whyte and Myers, 2009; Thonnard et al., 2013) have now been conducted to further examine this hypothesis. Whyte et al. (2005) found that in a cohort of 84 participants who had suffered a brain injury of various etiologies at least 4 months prior, only 04 or 05 percent cases demonstrated a perfect reaction on the CRS-R, demonstrating an enhanced consciousness level that occurred normally 01 to 02 h after administration but did not last for a prolonged period of time. For the second time, the Liège group described that only 12 patients (20 percent) displayed significant improvement in behavior and/or CRS-R, with only 01 individual showing important development as a result of functional object practice (Thonnard et al., 2013). This occurred in a sample of 60 persistent DOC patients (of whom 31 had TBI as the fundamental cause). Dopaminergic agonists (carbidopa/levodopa, bromocriptine, pramipexole) have only been researched in small-sample studies with a partial control, while dopamine receptor antagonists have only been studied in controlled investigations. They had some good benefits, but only to a certain extent. Sertraline is a selective serotonergic reuptake suppressor, was shown to be ineffective in improving the state of stimulation in a group of 11 individual who had had serious TBI (Meythaler et al., 2001).

Because of their minimal potential for injury, multi-sensory stimulation programs may also be a beneficial technique (Pape et al., 2015). In a preliminarily reported randomized, placebo-controlled trial evaluating the influence of accustomed aural sensory training in patients with dementia of the cerebral palsy, Pape et al. (2015) observed important improvements in the Coma-near-coma scale, which measures stimulation and attentiveness in individual with DOC. Two studies using multimodal sensory programs (Megha et al., 2013; Cheng et al., 2018) produced results that were similar to one another. According to the findings of the latter investigation, impact on the CRS-R, including enhanced provocation and oromotor functions, were just observed in MCS sick people and not in VS (Frazzitta et al., 2016; Cheng et al., 2018).

Moreover, given the unique characteristics of this group, it is fair to offer treatment in a specialized facility. The prevention and treatment of common problems are often the primary emphasis in these cases, in addition to the pharmaceutical choices and multi-sensory stimulation programs (Cheng et al., 2018) that have already been highlighted. It consists of measures to sustain dietary status and muscle mass, as well as measures to avoid and cure contractures and muscle spasms, as well as measures to keep the circulatory system functioning properly. According to one research, have a positive influence on clinical retrieval on the CRS-R (Frazzitta et al., 2016), while another study found no important variance in result between a conservative incline table and one with an assimilated treading maneuver (Krewer et al., 2015). The latter could be accomplished by frequent verticalization, which has been exposed to increase CRS-R recovery in one research (Frazzitta et al., 2016).

### Paroxysmal Sympathetic Hyperactivity

Even during the acute period of hospitalization, PSH might manifest itself. It can also be an issue in neurological rehabilitation settings. PSH is described as a recurring and episodic condition characterized by tachycardia, hypertension, diaphoresis, and hyperthermia (Baguley et al., 2014), among other signs and symptoms. Improved spasticity, dystonia, and extension or flexion posturing are all possible manifestations of this condition. It is possible that these symptoms are an excessive reaction to some painful external stimuli, for example suctioning of the endotracheal tube, sedation withdrawal, the patient’s passive manipulation, constipation, urine retention, or being in a very loud ambient environment. TBI (79.4 percent) is the most common cause of PSH, based on a systematic analysis by Perkes et al. (2010), trailed by hypoxic brain damage (9.7 percent) and stroke (0.1 percent; 5.4 percent). Although there are no stringent universal diagnostic criteria in the various research, it is impossible to compare the frequency of PSH in the different investigations. Because of this, while there is some agreement on the signs and symptoms of PSH, there has been a wide range of agreement on the number, duration, frequency, and severity of these symptoms among research.

### Posttraumatic Agitation

Posttraumatic agitation is a form of delirium that can provide a significant challenge in the treatment and reintegration of individual who have grieved a traumatic brain damage. When patients are in a state of emotional lability, they exhibit violent behavior, or disinhibition. According to the research, PA may occur in anywhere from 35 percent to 96 percent of instances of TBI during the acute phase of the injury. PA may last for a long time throughout the recovery period, and the agitated behavior has a significant impact on the rehabilitation. Lastly, it must be noted that the analysis of PA is a “diagnostic of exclusion,” meaning that it is made only after all therapeutic (metabolic, infection, and endocrine disorders, discomfort, and so on) and neurologic (for instance, hydrocephalus, migraine, cerebral lesion) reasons have been eliminated (Eapen et al., 2015). It is often suggested to decrease stimuli from the environment by reducing the amount of noise as well as the number of people; in addition, patients must be put in a peaceful padded room, on a vail bed, or on a netbed to reduce the amount of stimulation they get. Patients may also need one-on-one observation in certain cases. Furthermore, it is beneficial in removing unwanted or possibly tender stimuli for example tubes or catheters from the patient. It can be possible to lessen the patient’s cognitive confusion by keeping constancy among the reintegration team and by communicating in a concise and straightforward manner. The majority of the time, pharmaceutical intervention is required for agitation and neurobehavioral disorders. For the therapy and control of aggressiveness, a large number of medications has been tested, and various authors have published treatment recommendations (Warden et al., 2006; Luauté et al., 2016).

Luauté et al. (2016) conducted a meta-analysis of relevant studies on neuroleptic, antidepressants, mood stabilizers, beta-blockers, and drugs for the treatment of mood swings, aggression, irritability, and other neurobehavioral disorders, including schizophrenia. They reviewed 89 papers encompassing a total of 1306 person who had had a TBI. Ultimately, they determined that there was insufficient data to standardize medication treatments for these illnesses. Propranolol has been shown to reduce aggressiveness (evidence B grade). Agitation and aggressiveness are treated with carbamazepine and valproate, which are considered first-line medications for agitation and aggression (expert consensus opinion). Neuroleptics, which may be used in emergency circumstances, were not shown to be effective, according to the researchers.

Finally, according to a Cochrane Review (Fleminger et al., 2006), non-selective beta-blockers (pindolol and propranolol) had the greatest effectiveness for the management of PA, whereas other medicines failed to give conclusive evidence of their usefulness in the treatment of PA.

The effects of treatment should be assessed on a frequent basis, as well. It is advised that doctors conduct clinical evaluations on a regular basis. Nursing personnel may be assisted in assessing the clinical course of agitation by using simple measures for example the Richmond Agitation Sedation Scale (RASS) (Sessler et al., 2002), which is easily used. It was initially established to measure agitation and the impacts of sedative medicines in CCU individual, and it is therefore suitable to TBI individual as well (Robinson et al., 2019). However, to our information, it has never been evaluated sequentially in neurorehabilitation patients before. It is comprised of a 10-point scale that allows for the account of patient behavior ranging from unarousable (5) to confrontational (+4) in intensity. In addition, the Scale of Agitated Behavior (Bogner et al., 2000), which was designed expressly for use in TBI, provides a more thorough evaluation. It provides for a more complete evaluation of agitation and often requires a 10-min period of observation time. It comprises of 14 items that describe various behaviors, with a scoring scale ranging from 1 to 4 assigned based on the existence of the stated behavior and the intensity of the behavior.

### Posttraumatic Hydrocephalus

Posttraumatic hydrocephalus (PTH) is a progressive chronic condition characterized by increased cerebrospinal fluid (CSF) accumulates as a result of liquorodynamic changes following craniocerebral damage. The prevalence of PTH varies from 0.7 to 86 percent). Distinctions in diagnostic criteria and categorization have led to the disparity in stated prevalence (Choi et al., 2008).

Posttraumatic hydrocephalus is the most prevalent curable consequence during the course of recovery process after a traumatic brain damage (Guyot and Michael, 2000). The prevalence of symptomatic PTH varies from 0.7 percent to 29 percent (Mazzini et al., 2003). Alterations in diagnostic criteria and categorization have all attributed to the extensive list of stated prevalence rates that have been observed. The diagnosis of PTH is recognized by the use of arrangement of medical, radiological, and physiological conditions.

Communicating PTH and non-communicating PTH are the two forms of PTH that may be distinguished. It is known as communicative hydrocephalus because the various components of the ventricular system are linked and cerebrospinal fluid (CSF) moves from the ventricular system to the subarachnoid space in this condition. When a person has a TBI, communicating hydrocephalus is the most common kind, involving blood products or fibrosis obstructing the CSF passage into the circulation *via* the granulations of arachnoid. This can also apparent as what is known as common pressure hydrocephalus in certain cases (NPH). Obstructive hydrocephalus or non-communicating is characterized by the obstruction of CSF flow from flowing between the ventricles or leaving the ventricular system in the brain.

PTH is associated with cerebral hemorrhage (especially intraventricular hemorrhage), subarachnoid hemorrhage (SAH), the patient’s condition after decompressive craniectomy, meningitis, the length of time the patient has been in a coma, and the patient’s advanced age (Mazzini et al., 2003).

It is possible that a lack of recovery during early therapy, which is inconsistent with the severity of the injury, is a very early symptom of developing PTH. PTH, if left untreated, may result in clinical deterioration and a bad prognosis. This condition must be separated from posttraumatic ventriculomegaly caused by secondary atrophy, which is another form of PTH. Patients suffering from symptomatic PTH are likely to benefit from shunting treatment.

Kammersgaard et al. (2013) prospectively tracked 444 patients with serious TBI who needed long-term restoration. They found that 14.2 percent of the patients had PTH, with 75 percent of the instances developing throughout the reintegration process. Patients who had PTH were often older, had more serious TBI, were more commonly in VS, and required a lengthier reintegration stay than those who did not. A more detailed analysis revealed that older age and a lower degree of awareness were both linked with PTH when adjustments were made. According to the findings of this research, PTH is a problem that occurs mostly during in-patient rehabilitation. Conclusion: This complication of TBI should be monitored away from the acute stage, especially in elderly patients and those with significant impairment of consciousness, according to the authors’ findings.

According to the findings of a new cohort research with retrospective comparison investigation (Weintraub et al., 2017), out of 701 individuals hospitalized with TBI, 59 (8 percent) were found to have PTH. During rehabilitation, 36 individuals with PTH were able to exit PTA. This is a 61 percent success rate. Finally, they found that shunting was performed sooner in the process indicated a better success following rehabilitation.

Tian et al. (2008) conducted a study to determine the prevalence of PTH in individuals who had had traumatic SAH. The researchers found PTH in roughly 12 percent of the patients within 3 months of their hospitalization, with the bulk of cases occurring within 2–4 weeks following SAH. It has been reported that the emergence or finding of PTH might take up to 09 to 12 months after a TBI (Denes et al., 2011).

Non-communicating hydrocephalus is characterized by the existence of early signs of elevated intracranial pressure for example nausea, lethargy, vomiting, altered mental state, headaches, gait problems, and papilledema.

Secondary read NPH manifests as the triad of gait, urine incontinence, and dementia as the idiopathic type, with gait injury being the most possible to respond to surgical intervention. Poor activity introduction, psychomotor slowness, diminished attention, and amnesia are some of the cognitive impairments that might occur. Most typically, ventriculoperitoneal shunts (VPS) are used in the treatment of posttraumatic NPH. According to a study of the literature (Daou et al., 2016), the majority of patients who have shunt implantation will see clinical and radiographic improvement. In spite of the fact that the results of neuroimaging indicate a constellation consistent with NPH, the usual signs and indications of NPH may be obscured by the TBI consequences. Despite the fact that it seems logical to contemplate shunt implantation in this case, there is no compelling data to support this. The results of a retrospective study of 31 patients revealed that 65 percent saw significant development; a NPH that is not as severe as determined by neuroimaging and a younger age appeared to be associated with better results (Wen et al., 2009).

### Traumatic Brain Injury Patients With Posttraumatic Neuroendocrine Abnormalities

Neuropsychiatric problems are prevalent after TB and adversely affect TBI consequences by lowering overall standard of living. Historically, the emergence of neurobehavioral ramifications for instance, attention impairments, sadness, anxiety, exhaustion, and a reduction of emotional well-being has been ascribed to an undefined “post-concussive syndrome” thought to be related to severe structural damage and axonal injury. Latest investigation, however, indicates that neuroendocrine disorder, notably hypopituitarism, is a significant contributor to the genesis of these symptoms (Molaie and Maguire, 2018).

Finally, throughout the course of neurorehabilitation after a TBI, one may be presented with neuroendocrine abnormalities as a result of pituitary lesions. They are often overlooked and unappreciated for their contributions. Pituitary gland injuries have been linked to acceleration–deceleration injuries (Webb et al., 2014). This is likely owing to the susceptibility of the pituitary gland’s vascular supply *via* the infundibulum, as well as its strong encasement inside the sella turcica. Although more skeptical, Klose and Feldt-Rasmussen hypothesize that the frequently observed changes in anterior pituitary hormones following TBI are quite parallel to pituitary changes in other serious illnesses and should be measured as a physiologic alteration to serious stress, as it frequently instinctively concerns (Klose and Feldt-Rasmussen, 2018). As an example of this, Agha et al. (2004) reported an 18 percent prevalence of growth hormone shortage, a 16 percent prevalence of adrenocorticotropic hormone insufficiency, 52 percent hyperprolactinemia, diabetes insipidus, or 40% have either type of improper antidiuretic hormone syndrome, and an appreciable decrease in serum thyroid-stimulating hormone.

The research on hypopituitarism in the chronic condition of TBI is not consistent, and research have found incidence rates ranging from 0 to over 70 percent (Klose and Feldt-Rasmussen, 2018) in patients with TBI. The German Interdisciplinary Database, which was created in 2005, comprises a nationwide register of people who have endured TBI or injury of spinal cord. By means of the Structured Data valuation of Hypopituitarism after TBI and SDAH, a total of 1242 patients were incorporated in the initial publication (Schneider et al., 2011). Physician diagnoses, laboratory values, and stimulation tests all revealed that the incidence of the chronic phase hypopituitarism (at least 05 months after the occurrence) was 35 percent, 36 percent, and 70 percent, respectively, in the chronic phase. Persons with TBI who underwent aberrant inspiration tests had had more serious TBI than individual who underwent typical activation tests. TBI and SAH are both known to cause hypopituitarism, which the authors determined is a frequent consequence. A follow-up publication (Krewer et al., 2016) on patients who had been observed for 1–5 years or more found that the maximum incidence of neuroendocrine ailments occurred 1–2 years after the damage, and that the prevalence reduced over time before reaching a new high point in the long process in patients who had experienced a brain damage less than 5 years before the assessment. Gonadotropic deficiency was the most prevalent hormonal imbalance in the subgroup of individuals who had suffered injury of brain between 1 and 2 years (*n* = 126), trailed by somatotropic inadequacy (11.5 percent), corticotropic inadequacy (9.2 percent), and thyrotropic inadequacy (7.3 percent; 3.3 percent). Patients with fewer than 5 years of observation time following brain damage had a higher incidence of somatotropic insufficiency, which climbed to 24.1 percent over time, but corticotropic and thyrotrophic insufficiency became less common over the same period (2.5 percent and 0 percent, respectively). Neuroendocrine abnormalities are common even years after a TBI or a stroke, according to findings from a cohort of patients who are still receiving medicinal therapy (Krewer et al., 2016).

**FIGURE 3:**
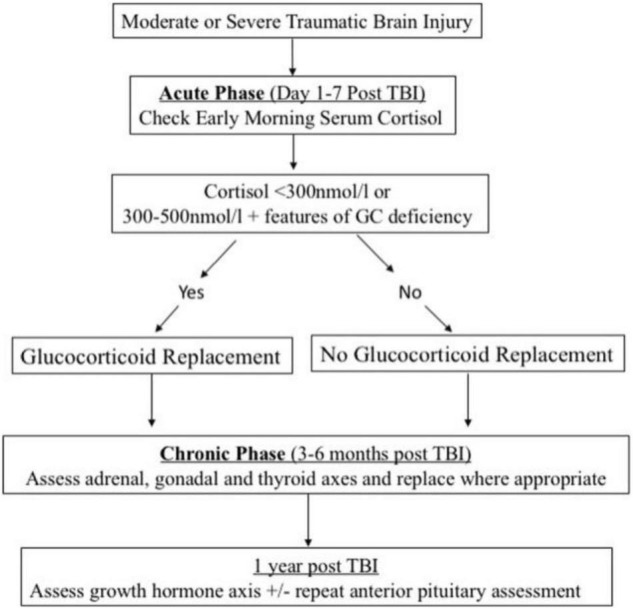
Screening and management algorithm for post-traumatic hypopituitarism. Reproduced with permission from Quinn and Agha (2018).

Hypopituitarism can manifest itself in separate clinical manifestations, particularly in a cohort of severely impacted individuals, and testing laboratory looks to be unpredictable during the acute stage (Klose and Feldt-Rasmussen, 2018). As a result, there is disagreement regarding regardless of whether research lab screening or activation tests must be conducted on a regular basis. When it comes to the acute phase, Klose and Feldt-Rasmussen came to the conclusion that current evidence did not support regular testing of the thyroid, GH, and gonadal axis, but that prompt care was necessary when antidiuretic and adrenal hormone deficiency was medically supposed (Klose and Feldt-Rasmussen, 2018). According to other researchers (Quinn and Agha, 2018), screening for ACTH insufficiency should be performed in patients with moderate to severe TBI since low levels of plasma cortisol are a predictor of death in another research (Figure 3; Hannon et al., 2013). They believe that recurrent morning cortisol levels of less than 300 nmol/L are indicative of adrenal inadequacy, necessitating the use of a corticosteroid substitute (Quinn and Agha, 2018). Nonetheless, to our knowledge, no large-scale investigations have shown a direct association between pituitary dysfunction and higher mortality in the general population.

## Admittance to Institutions After TBI

### Who Will Be Admitted to Neurorestoration?

Rates of admission to neuroreintegration for the victims of serious TBI vary substantially from country to country. In specialized trauma centers in Texas (11 centers in the United States), Norway 4 centers), (Switzerland 12 centers), and Denmark (4–5 centers), rates of 45 percent, 44 percent (+16 percent non-specialized rehabilitation), 75 percent (+11 percent non-specialized rehabilitation), and 84 percent have been reported, respectively (Shafi et al., 2014; Schumacher et al., 2016; Sveen et al., 2016). Inequality in the populations (i.e., the Danish and Norwegian populations have a significantly lower mean age than the United States and a significantly higher proportion of younger patients than the United States) limits the comparability of the data, but these variances might also be described in part by factors inherent to the health-care system, for example national rules, institutions and expense coverage/reimbursement.

### What Are the Admissions Requirements?

Greenwald and Rigg (2009) published a review in which they gave the following conditions for in-patient recuperation, which were apparently not constructed on confirmation but rather developed from practical knowledge. If the patient demonstrates the ability to learn/change that inhibits him or her from coming back home with caregivers, if the patient’s surgical and medical circumstances are extremely stable to permit involvement in treatments, if the individual exhibits the capability to engage in at least 1 h of treatment twice a day, if the individual illustrates the capability to grow in acute care treatments, and if the individual has access to a social support network which would permit to come home with caregivers.

To put it more simply, the Canadian INESSS-ONF “Clinical Practice Guideline for the Rehabilitation of Adults with Moderate to Severe TBI” (Lamontagne et al., 2016) states which “Rehabilitation programs should have clearly stated admission criteria, which should include a TBI diagnosis, medical stability, the ability to improve through the rehabilitation process, the ability to learn and participate in rehabilitation, and sufficient tolerance for therapy duration.”

### Regarding Current Practice, Which Patients Should Be Excluded and Which Should Not Be?

Traumatic brain injury patients over the age of 65 are often refused access to specialist rehabilitation (Cnossen et al., 2017). According to the findings of our group’s extensive evaluation of the literature, elder patient having TBI have the ability to make considerable gains in the long run, even if their initial outcomes are less favorable than those of younger patients (Schumacher et al., 2017).

When it comes to practical considerations, the most common causes for exclusion from reintegration are premorbid conditions that either significantly impair the reliance on care prior to the TBI, such as in severe dementia, or a palliative condition owing to progressive human cancers, are both related with a poor long-term outcome.

## The Impact of Timing and Rehabilitation Programs on Traumatic Brain Injury Rehabilitation Outcomes

There are presently no worldwide commendations for the treatment of individuals with serious (TBI) during the early recovery period. Only a few research have looked examined the effects of incorporating recuperation into acute TBI therapy.

A Randomized review investigating at comprehensive rehabilitation for people of working age who had suffered brain impairment (Turner-Stokes et al., 2015) found that intense therapy seems to result in greater improvements sooner after the injury has occurred. There are only a few investigations that demonstrate the efficacy of early interference in emergency and acute care settings. Patients who need cognitive rehabilitation after a serious brain damage benefit from group-built therapy in a healing setting (in which sick people go through cognitive reintegration in a healing setting alongside an individual’s peer group who have been exposed to similar contests). The authors stressed that RCTs (randomized controlled trials) and other experimental methodologies are not always appropriate for addressing all concerns in rehabilitation. The pilot-based texts does not provide answers to the questions of which therapies are most effective for patients in the long run, or reintegration facilities deliver the most value for cash in the milieu of long-term care and restoration.

According to Turner-Stokes in another review study (Turner-Stokes, 2008), the published evidence on the efficacy of multimodal reintegration after developed brain damage in individuals of operational age has been thoroughly examined. It was determined that the best evidence was obtained from an RCT and when compared matched to the literature produced for the United Kingdom Long-Term Neurological Ailment National Service Framework, by means of a typology built on assessment in terms of research reliability regardless of study methodology.

She underlined that the reintegration of TBI patients is a complicated procedure that presents some hurdles for medical investigation that tend to contradict typical randomized controlled trials (RCTs). Because it is not applicable to all of the problems that need to be addressed in TBI rehabilitation (Turner-Stokes, 2008), it is difficult to rely only on the randomized controlled trials technique in rehabilitation research. The previous rehabilitation studies concludes: as a result, there is significant variation across studies in terms of TBI severity, the intervention used and the clinical context in which it was conducted. Furthermore, the effects may fluctuate depending on the healing process. The ethical implications are important because many individuals with moderate to serious TBI can shortage the intellectual ability to provide completely knowledgeable permission for study participation. Furthermore, given the growing body of data demonstrating the efficacy of multidisciplinary rehabilitation in a variety of illnesses (especially stroke), it is immoral to assign individual to “no therapy” or even “standard” care in clinical trials. The time span over which recuperation can have an impact (months or years) is often significantly greater than the time span over which any financed research study may be completed. They used the GRADE system suggested by the GRADE Working Group to evaluate the effectiveness of rehabilitation for adults with TBI (Atkins et al., 2005). They looked at both randomized controlled trials and non-randomized controlled trials to support the efficiency of restoration for grown person with TBI. Table 1 lists the suggestions for clinical practice that have been developed.

**TABLE 1:** Various neurorehabilitation techniques in TBI are recommended for clinical practice (that use the GRADE system).

Evidence of quality	Rehabilitation	Kind of patients	Results	Possible of cost savings	Commendation (GRADE System)
High	Severe	Severe TBI	Increase independence and decreased duration of hospitalization	+	Recommended strongly
Moderate/high	Specialist	TBI that is very severe/serious	Improve individuality and reduce continuing treatment	+ +	Recommended
	Specialist vocational programs	Moderate/severe TBI	Improve efficiency	+ +	Recommended strongly
Moderate	Primary	Serious TBI	Increase individuality decreased hospital LOS	+	Recommended
	Community based	Moderate/severe TBI	Productivity enhancements	_++_	Recommended
Low/moderate	Behavioral management programs	TBI with severe behavioral complications	Enhanced social skill and decreased long-term care	+	Recommended
	Late and ongoing rehabilitation	Moderate/severe TBI resulting in long-term impairment	Retaining independence and efficiency	+	Recommended conditionally

*GRADE, Grading of Recommendations Analysis, Improvement, and Assessment; TBI, traumatic brain injury). + Represent saving cost onefold, and ++ represent saving cost twofolds.*

These findings provide a strong argument for for more intense reintegration services in serious TBI, which are linked with quicker functional improvements. They found significant evidence in support of this advice. There is moderate to strong evidence in support of specialized or vocational rehabilitation programs, but there is only moderate evidence in support of programs that continue outpatient treatment and in support of early intervention programs.

More recent research, such as that conducted by Andelic et al. (2012), has provided support for the notion of extremely early rehabilitation. They investigated if constant network of rehabilitation beginning in the severe stage of retrieval from severe TBI enhances the functional status among these patients as associated having a wrecked series of reintegration that begins in the subacute stage of recovery from thirty-one TBI individuals in the initial restoration category and thirty individuals were in the late reintegration group—in a cohort of 61 trying to survive individuals with serious TBI. The GOSE and DRS were used to evaluate the outcome 12 months resulting a TBI. A statistically important satisfactory result (GOSE 6–8) happened in seventy one percent of the sick people in the primary reintegration category compared to 37 percent of the patients in the late rehabilitation group. A similar result was obtained in relation to the DRS score, which was considerably higher in the initial recovery group. According to the findings of a recent review, the presented data shows that primary initiation of neurorehabilitation in a trauma center, as well as more intensive neurorehabilitation in the post-acute context, increase efficient rehabilitation (Königs et al., 2018).

Rehabilitation facilities are usually situated in different locations than acute care facilities, and they do not have the resources to provide rigorous monitoring for patients who are experiencing initial therapeutic issues (for instance, PA, CS/PTA, PSH). Because of this, it seems that the time gap between the development of brain damage and admission appears to be mostly dependent on the degree of brain damage and any concurrent difficulties in these typical settings (Formisano et al., 2017).

## Multiprofessional In-Patient Restoration Duration and Intensity

### Intensity

It is difficult to provide an answer to the topic of the ideal intensity of treatments in neurological restoration due to the variety of individuals. Nonetheless, some studies revealed indications of early improvements in TBI patients who received more intense treatment. In the first study, Zhu et al. (2001) linked more rigorous (04 h per day) treatment to standard (02 h per day) reintegration in 68 sick people. A considerably larger percentage of individuals enrolled in a more intense program achieved their maximum GOS scores in 3 months’ time, with no important differences seen at any other point in time throughout the study. Shiel et al. (2001) conducted a research in which they compared “regular” multidisciplinary therapy to “high intensity” multidisciplinary rehabilitation in two research centers with a total of 51 patients. Comparing the augmented intensity group to the control group, they found that the functional scores (FIM + FAM) improved by a statistically significant amount (therapy doses are not indicated in relative or absolute terms).

In comparison, Hart et al. (2016) looked at consequences 1 year following serious 274 patients with TBI from 02 dissimilar research areas in the US and 01 in Denmark—and found no significant differences. However, despite the fact that the Danish site supplied considerably more intensity and frequency of regeneration, there were no area distinctions in emotional and functional objectives at 12 months; it was due to the Danish population are seriously impacted; and thus they adjusted the patient/injury features. It is tricky hard to draw generalizations from this research, although it was not possible to demonstrate clean strength and/or duration connected benefits in this study.

Nonetheless, in our clinical experience with patients who have suffered a severe TBI, they might have a limited capacity to engage in extended treatment sessions as well as in group therapies with high fatigability in the 1st few weeks after the damage. This has the potential to be a significant restraining factor for greater treatment intensities.

### Duration

In one sense, more rigorous treatment may have the potential to shorten the length of stay. With regard to multi-disciplinary rehabilitation, Slade et al. (2002) evaluated two groups that received varying intensities of care, with one group benefiting from 30 percent more treatment time than the other. The results of a multiple regression model following statistical adjustment for confounding variables (community delays, impaired mix, missed treatment) showed that the group receiving more intensive treatment had a 14-day shorter length of stay (LOS) than the group receiving less intense therapy and functional scores that are equivalent (Barthel index). A similar finding was seen in Shiel’s (Shiel et al., 2001) data, where the LOS was dramatically decreased in one group that received more extensive therapy, but only at one research location. In contrast, the authors note that the LOS was significantly longer in the second research site because of a disproportionately large number of patients who were very badly injured.

However observational studies (McLafferty et al., 2016; Formisano et al., 2018) reported that extended rehabilitation duration are linked with more significant enhancements in DRS or FIM scores, as determined by the FIM or DRS, respectively. On the other hand, Foy and Somers were able to establish a link between LOS and functional gains in young people who were engaging in a program for inpatients that included 5 h of treatment and/or education each day. Despite the fact that this cannot be converted into straightforward LOS-related gains, it might signal that patients who made less significant progress throughout rehabilitation were discharged early in those trials, which is a possibility in theory. On the other hand, they determine that restoration in-patient therapies that could last for more than 04 months individuals having chronic neurological disorders (However, only 18 percent of the sample had TBI) is economical because it reduces dependency and, as a result, the expense of long-term treatment in particular patients.

Length of stay in in-patient rehabilitation is influenced by a variety of variables in therapeutic settings, including: (i) Individual objectives and motivation, family and caregiver support, and employment prestige and position, to name a few examples; (ii) associated health system issues, such as predetermined maximum lengths of stay (LOS), restricted capability of reintegration facilities, accessibility of intense outpatient/community programs, and nursing/care home placement, to mention a few.

## Are There Any Rehabilitation Methods That Are Effective Than Others?

As stated by Shoulson et al. (2012) and Brasure et al. (2013), the Institute of Medicine and the available inadequate information does not permit for judgments on the comparative influence of various rehabilitation centers, such as in context of outcome metrics for activities and involvement, amongst other things. These results must be seen in the context of the challenges previously noted in relation to rehabilitation trials, which must be considered.

If Cicerone et al. (2008) were to demonstrate that an intense cognitive rehabilitation program is superior to ordinary therapy in terms of patient productivity, they would have established a precedent. Furthermore, although progress during rehabilitation is not always quantifiable *via* the use of scales, it may still be of tremendous benefit to the person. Cicerone et al. (2011) that there was sufficient indication to suggest intervention strategies for awareness, communication skills, executive function in social situations, memory, as well as total-holistic neuropsychological restoration after TBI in their review of scientific proof cognitive rehabilitation. According to the guiding principle, an international organization of academics and clinicians (INCOG) suggest a comprehensive intellectual reintegration facility customized to patient neurobehavioral profiles, premorbid intellectual features, and aspirations for life actions and contribution. Furthermore, according to the Scottish recommendation on adult brain damage recovery (British Thoracic, 2003), these measures should be included in detailed neuropsychological rehabilitation centers delivered by a multidisciplinary teams that used a goal-oriented scheme able to address behavioral, emotional, and cognitive problems with the ultimate goal of achieving working in important daily activities.

As a rule, an experienced team of doctors, neuropsychologists, physiotherapists, nursing team, occupational and speech therapists alternatively, it may also contain social workers, nutritionists, and therapists that specialize in recreational/vocational treatment. The team should meet on a frequent basis to examine the patient’s condition, progress, potential barriers to progress and to develop treatment. It is recommended that the effected personnel get frequent education to ensure that they understand and comply with the treatment plan as well as the potential results.

## Conclusion

Neurologists and other medical doctor who work in the TBI reintegration must be aware of the differences between moderate and severe TBI in terms of diagnostic methods, lesion patterns, and the distinctive series of recovery, along with the aforementioned common problems that can impede rehabilitation progress. Due to the wide range of behavioral, cognitive, physical, and psychosocial consequences of TBI, the authors’ experience and that of several other experts has led them to believe that rehabilitation must be individualized and concentrated on the patients’ needs, goals, strengths, and deficiencies as defined by the International Classification of Functioning, Disability, and Health [ICFDH]—in order to be effective (ICF). Furthermore, early rehabilitation might have a positive impact on the results of individual.

## Author Contributions

MA, UT, KT, MK, and MC acquired the data, analyzed and interpreted the data, drafted and critically revised the manuscript, contributed to study conception, design, and financial support. All authors contributed to the study while adhering to the ICMJE guidelines, reviewed and approved the submitted version of the work, agreed to be individually responsible for his or her own contributions as well as to ensure that any questions about the precision or integrity of any part of the work, even if the author was not involved directly, are adequately evaluated and resolved, and that the resolution is documented in the literature.

## Conflict of Interest

The authors declare that the research was conducted in the absence of any commercial or financial relationships that could be construed as a potential conflict of interest.

## Publisher’s Note

All claims expressed in this article are solely those of the authors and do not necessarily represent those of their affiliated organizations, or those of the publisher, the editors and the reviewers. Any product that may be evaluated in this article, or claim that may be made by its manufacturer, is not guaranteed or endorsed by the publisher.
